# Efficacy of ultrasound-guided percutaneous transhepatic biliary drainage for acute obstructive suppurative cholangitis combined with septic shock

**DOI:** 10.1016/j.clinsp.2023.100258

**Published:** 2023-07-29

**Authors:** Miao Meng, Hui Feng, Shuan Tang, Xiaobin Peng

**Affiliations:** Department of Gastroenterology, The Affiliated Wuxi n 2 People's Hospital of Nanjing Medical University, Jiangsu, China

**Keywords:** PTCD, AOSC, Septic shock, Inflammatory factors

## Abstract

**Objectives:**

This study aimed to investigate the efficacy of ultrasound-guided Percutaneous Transhepatic Biliary Drainage (PTCD) for the treatment of Acute Obstructive Suppurative Cholangitis (AOSC) combined with septic shock due to choledocholithiasis, and its effect on inflammatory factors.

**Methods:**

Clinical data of 86 patients with AOSC and septic shock admitted to our hospital between January 2019 and May 2021 were retrospectively analyzed and grouped according to different treatment methods. Among them, 43 patients who underwent Endoscopic Retrograde Cholangiopancreatography (ERCP) and Endoscopic Nasobiliary Drainage (ENBD) were included in the Control Group (CNG), and 43 patients who underwent ultrasound-guided PTCD were included in the Study Group (SG).

**Results:**

The total effective rate in the SG (88.37%) was higher than that in the CNG (69.77%) (*p <* 0.05); after surgery, the serum inflammatory factors PCT, IL-6, TNF-α, CRP levels, liver function indicators such as TBIL, DBIL, AST, ALT levels, and stress response indicators including NPY, PGE2, 5-HT levels were reduced, and were lower in the SG than in the CNG (*p <* 0.05); postoperatively, CD3+, CD4+, and CD4+/CD8+ levels in the CNG were significantly lower than those before surgery (*p <* 0.05); Postoperatively, CD3+, CD4+, and CD4+/CD8+ levels in the SG were significantly higher than those in the CNG (*p <* 0.05); and the complication rate in the SG (6.98%) was lower than that in the CNG (25.58%) (*p <* 0.05).

**Conclusions:**

Ultrasound-guided PTCD for AOSC combined with septic shock can facilitate the recovery of liver and immune functions with a low complication rate.

## Highlights

• Ultrasound-guided PTCD improves treatment effect for AOSC combined with septic shock.
• Ultrasound-guided PTCD reduces stress response for AOSC combined with septic shock.
• Ultrasound-guided PTCD assists patients smoothly to pass preoperative critical period.

## Introduction

Acute Obstructive Suppurative Cholangitis (AOSC) is induced by biliary obstruction and infection and is one of the most common acute abdominal diseases and a primary cause of septic shock. AOSC with septic shock can manifest with high fever, abdominal pain, chills, jaundice, decreased blood pressure, restlessness, apathy, and delirium. This condition is critical, and respiratory distress, renal failure, and multiple organ failure are likely to occur. The morbidity and mortality rates are as high as 13%–88%.^1^^,^^2^ Currently, treatment options for patients with AOSC and septic shock include active anti-shock and antibiotics, early biliary decompression, bile drainage, and the release of obstruction.^3^ In the past, surgery was the main treatment option for AOSC, including a common bile duct incision with pus drainage to remove gallstones and relieve biliary obstruction; however, it was found that patients with AOSC combined with septic shock had morbidity and mortality rates ranging from 20% to 40%.^4^

With the development of endoscopic technology, ERCP and ENBD have been gradually applied clinically at an early stage, which can effectively reduce pressure and unblock the draining bile ducts. However, endoscopic treatment requires high technical skills and is more painful for patients.^5^ PTCD is a procedure that relieves the symptoms of obstructive jaundice and causes less trauma, which can rapidly relieve critical condition, correct shock, and reduce jaundice.^6^ This study investigated the effects of ultrasound-guided PTCD in the treatment of AOSC combined with septic shock caused by choledocholithiasis.

## Materials and methods

### General data

The clinical data of 86 patients with AOSC and septic shock caused by choledocholithiasis, who were admitted to our hospital between January 2019 and May 2021, were retrospectively analyzed. All patients met the following inclusion criteria of “Guidelines for the diagnosis and treatment of acute biliary system infection (2021 edition)”^7^ and “Chinese clinical practice guidelines for emergency infectious shock”:^8^ (1) Accompanied by acute abdominal pain, jaundice, high fever and chills, and shock manifestations such as decreased blood pressure, irritability, apathy, and delirium; (2) Laboratory examination of leukocyte count ≥ 20 × 109 L; (3) Imaging examination confirmed the diagnosis of AOSC caused by choledocholithiasis and showed dilated intra- and extrahepatic bile ducts. Patients with the following conditions were excluded: (1) Combined portal hypertension, severe esophageal varices, or liver disease; (2) Combined hepatocellular carcinoma, gastric cancer, pancreatic cancer, or bile duct cancer; (3) Combined coagulation or hematologic disorders or abnormal immune function; (4) Combined abnormal cardiopulmonary function or other systemic infections; and (5) A history of upper abdominal surgery. Patients were grouped according to the different treatment methods: 43 patients who underwent Endoscopic Retrograde Cholangiopancreatography (ERCP) and Endoscopic Nasobiliary Drainage (ENBD) were included in the Control Group (CNG), and 43 patients who underwent ultrasound-guided PTCD were included in the Study Group (SG). In the SG, there were 25 males and 18 females; their ages ranged from 58 to 78 years, with a mean age of 67.74 (± 5.98) years; the diameter of the common bile duct ranged from 0.86 to 1.87 cm, with a mean diameter of (1.48 ± 0.13) cm. In the CNG, there were 26 males and 17 females; aged 58‒78 years, mean age of (67.79 ± 5.97) years; the diameter of the common bile duct was 0.85‒1.89 cm, mean of 1.49 (± 0.14) cm. The general data of the two groups were compared, and the differences were not statistically significant (*p* > 0.05). This study was approved by the Medical Ethics Committee of the Affiliated Wuxi nº 2 People's Hospital of Nanjing Medical University (nº 2022BN-086). The patients' families signed an informed consent form. The procedures of this study followed CONSORT Statement.

## Methods

After admission, all patients were actively treated with symptomatic support such as dietary suppression, antibiotics, anti-shock measures, rehydration, and nutritional support. Within 24 h of admission, the CNG was treated with ERCP and ENBD, and the SG was treated with ultrasound-guided PTCD.

ERCP was performed with the patient in the left lateral position with an intravenous injection of scopolamine 10‒20 mg + diazepam 5‒10 mg + pethidine 25‒50 mg. ERCP was performed by advancing the duodenoscope into the descending segment of the duodenum, incising the duodenal papillary sphincter under direct visualization, and inserting a guidewire into the bile duct. Bile was drawn, the contrast agent was slowly injected, and a nasobiliary drainage tube was placed simultaneously.

### Ultrasound-guided PTCD

In the left lateral recumbent position, the dilated intrahepatic bile duct was explored using ultrasound; a dilated intrahepatic bile duct with a large diameter (≥ 4 mm) and a straight course was taken as the target bile duct, and the 7th‒9th intercostal space in the mid-axillary line or under the saber was taken as the puncture point. After local infiltration anesthesia with 2% lidocaine, a 4- to 5 mm incision was made, and a puncture needle was inserted into the target bile duct through the incision under ultrasound guidance. The needle core was removed, the guidewire was inserted after the purulent bile had flowed out, the drainage tube was inserted along the guidewire into the bile duct cavity after the expansion tube was expanded, the guidewire was pulled out, and the drainage tube was unobstructed, fixed on the patient's skin, and connected to the drainage bag. Both groups continued to receive rehydration, hemostasis, antibiotics, anti-shock measures, and other postoperative treatments.

### Outcome measurement

(1) General surgical outcomes and relief of clinical symptoms were recorded: the operation time, intraoperative blood loss, duration of fever, chills, abdominal pain, and drainage tube retention were recorded in both groups. (2) Evaluation criteria for efficacy: Cure: clinical symptoms such as fever, chills, and abdominal pain disappeared within seven days, and routine blood results, inflammatory factors, and liver biochemical indices were normal; markedly effective: clinical symptoms disappeared or remitted within 10 days, and routine blood tests, inflammatory factors, and liver biochemical indices were generally normal; effective: clinical symptoms remitted within 14 days, and routine blood results, inflammatory factors, and liver biochemical indices improved significantly; ineffective: symptoms did not remit or even worsen after 14 days. The total effective rate = (cure + markedly effective + effective)/ number of cases ×100. (3) Inflammatory factor: 5 mL of venous blood was collected before and three days after surgery, centrifuged at 3000 r/min for 10 min, and the serum was extracted. The levels of Calcitoninogen (PCT), Interleukin-6 (IL-6), Tumor Necrosis Factor-α (TNF-α) and C-Reactive Protein (CRP) were detected by electrogenerated chemiluminescence. (4) Liver function: 5 mL of venous blood was collected before and three days after surgery, and Total Bilirubin (TBIL), Direct Bilirubin (DBIL), Glutathione Aminotransferase (AST), and glutamic acid Aminotransferase (ALT) levels were measured using a biochemical analyzer. (5) Stress response: 5 mL of venous blood was collected before and three days after surgery and centrifuged at 3500 r/min for three min, and the levels of Neuropeptide (NPY), Prostaglandin E2 (PGE2), and 5-Hydroxytryptamine (5-HT) were measured by enzyme-linked immunosorbent assay. (6) Immune function index levels: 5 mL of venous blood was collected before and three days after surgery; CD3+, CD4+, and CD8+ levels were measured by flow cytometry, and CD4+/CD8+ was calculated. (7) Complications: Complications occurring within 14 days of surgery, including incisional infection, trocar obstruction, acute pancreatitis, biliary fistula, and gastrointestinal bleeding, were recorded.

### Statistical methods

SPSS software (version 21.0) was used for statistical analysis, and the measurement data were expressed as $\bar{x}\pm s$and examined using a *t*-test. The count data were expressed as a rate (%) and examined with χ^2^ *test.* Differences *were* considered statistically significant at *p <* 0.05.

## Results

### General surgical outcome and clinical symptom relief

The operating time, intraoperative blood loss, duration of fever, abdominal pain, chills, and drainage retention in the SG were shorter in the SG than in the CNG (*p <* 0.05, Table 1).

**Table 1:** Comparison of general surgical outcome and symptom relief ($\bar{x}$±s).

Group	n	Operative time (min)	Intraoperative blood loss (mL)	Duration of fever (d)	Duration of Chills (d)	Duration of Abdominal pain (d)	Duration of indwelling drainage tube (d)
Study group	43	95.46 ± 9.47	28.64 ± 2.75	2.07 ± 0.20	1.76 ± 0.17	7.64 ± 0.75	8.73 ± 0.84
Control group	43	118.61 ± 11.86	49.52 ± 4.91	2.46 ± 0.23	1.98 ± 0.18	8.95 ± 0.89	10.62 ± 1.05
*T*		10.002	24.330	8.391	5.827	7.381	9.217
p		0.000	0.000	0.000	0.000	0.000	0.000

### Comparison of clinical efficacy

The total efficacy rate of the SG (88.37%) was higher than that of the CNG (69.77%) (*p <* 0.05, Table 2).

**Table 2:** Comparison of clinical efficacy (cases [%]).

Group	n	Cure	Markedly effective	Effective	Ineffective	Total effective rate
Study group	43	12 (27.91)	20 (46.51)	6 (13.95)	5 (11.63)	38 (88.37)
Control group	43	7 (16.28)	14 (32.56)	9 (20.93)	13 (30.23)	30 (69.77)
*χ^2^*						4.444
p						0.035

### Comparison of inflammatory factors

**Fig. 1:**
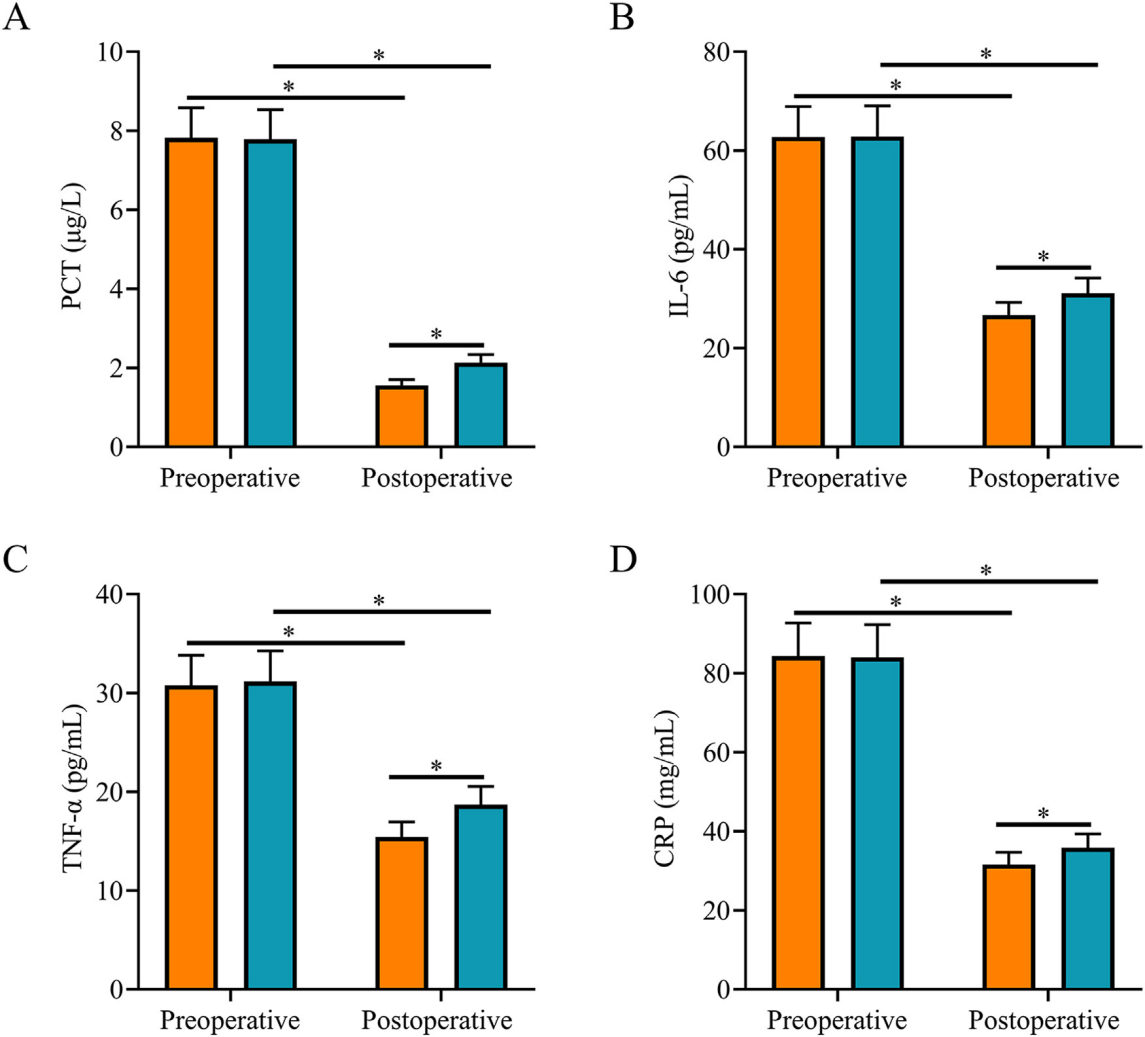
**Comparison of inflammatory factors.** (A) Postoperative serum PCT level in the study group was lower than that in the control group; (B) Postoperative serum IL-6 level in the study group was lower than that in the control group; (C) Postoperative serum TNF-α level in the study group was lower than that in the control group; (D) Postoperative serum CRP level in the study group was lower than that in the control group. Note: *p < 0.05.

After surgery, serum PCT, IL-6, TNF-α, and CRP levels were significantly lower in both groups, and they were all lower in the SG than in the CNG (*p <* 0.05, Fig. 1).

### Comparison of liver function index

**Fig. 2:**
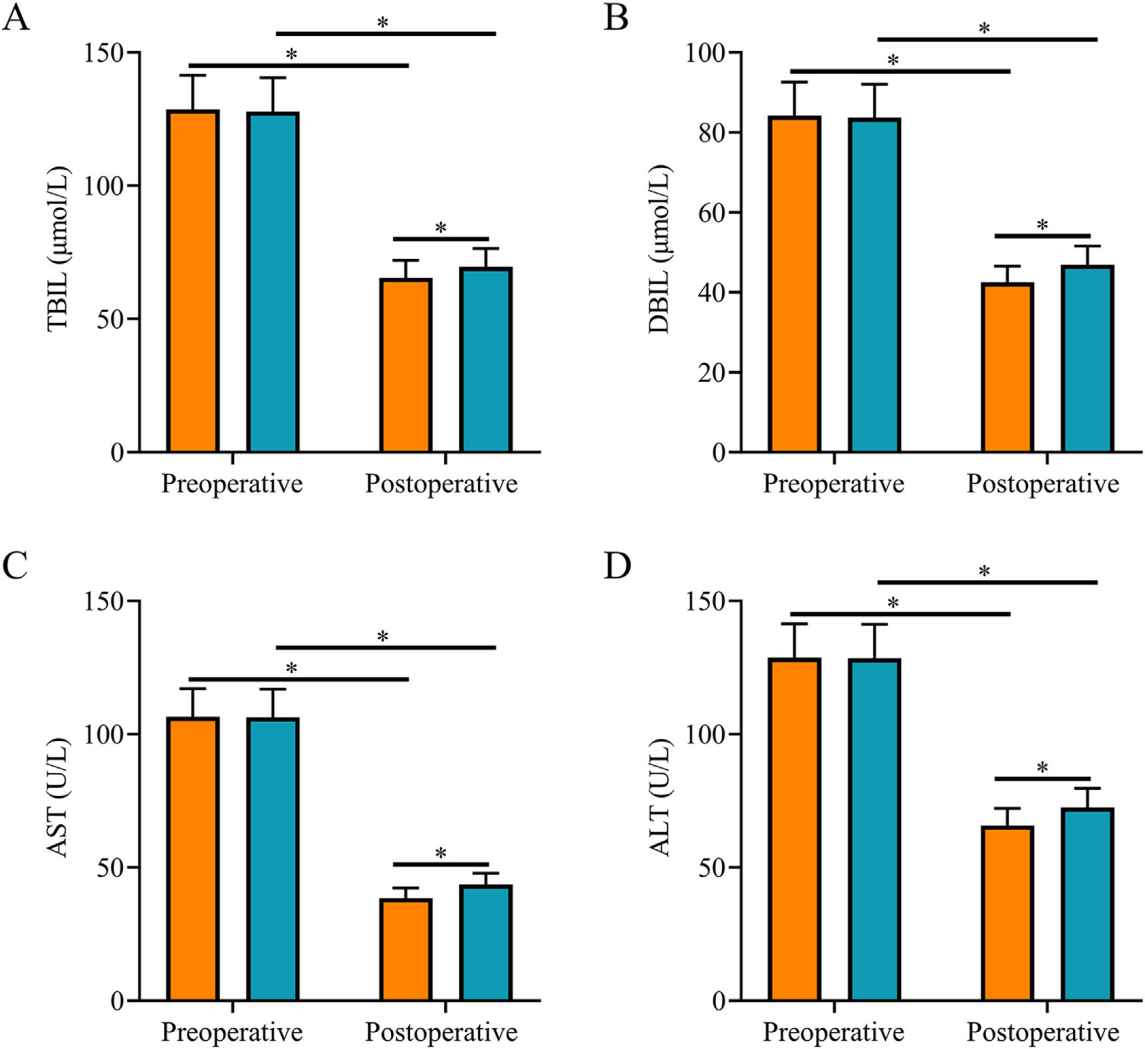
**Comparison of liver function indexes.** (A) The postoperative serum TBIL level in the study group was lower than that in the control group; (B) The postoperative serum DBIL level in the study group was lower than that in the control group; (C) The postoperative serum AST level in the study group was lower than that in the control group; (D) The postoperative serum ALT level in the study group was lower than that in the control group. Note: *p < 0.05.

After surgery, TBIL, DBIL, AST, and ALT levels were reduced in both groups and were all lower in SG than in CNG (*p <* 0.05, Fig. 2).

### Comparison of stress response indexes

**Fig. 3:**
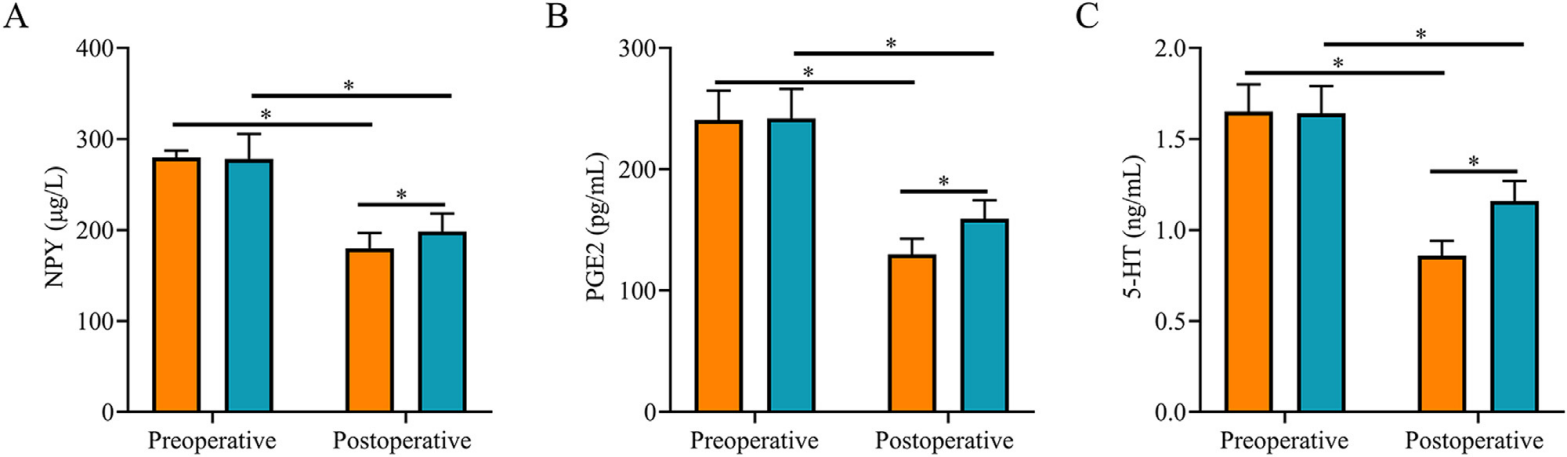
**Comparison of stress response indexes between two groups.** (A) The postoperative serum NPY level in the study group was lower than that in the control group; (B) The postoperative serum PGE2 level in the study group was lower than that in the control group; (C) The postoperative serum 5-HT level in the study group was lower than that in the control group. Note: * *p <* 0.05.

After surgery, serum NPY, PGE2, and 5-HT levels were reduced in both groups and were lower in SG than in CNG (*p <* 0.05, Fig. 3).

### Comparison of immune function

After surgery, there were no statistically significant differences in CD3+, CD4+, and CD4+/CD8+ levels before and after treatment in the SG (*p* > 0.05), while the CD3+, CD4+, and CD4+/CD8+ levels in the CNG were all significantly lower than those before treatment (*p <* 0.05); After surgery, the CD3+, CD4+, and CD4+/CD8+ levels were significantly higher in the SG than in the CNG (*p <* 0.05, Table 3).

**Table 3:** Comparison of immune function index ($\bar{x}$±s).

Group	n	CD3+ (%)		CD4+ (%)		CD4+/CD8+	
		Preoperative	Postoperative	Preoperative	Postoperative	Pre-operative	Postoperative
Study group	43	65.12 ± 6.48	62.84 ± 6.28	39.23 ± 3.90	37.96 ± 3.79	1.62 ± 0.16	1.56 ± 0.15
Control group	43	65.34 ± 6.50	57.71 ± 5.42^a^	39.18 ± 3.87	34.52 ± 3.43^a^	1.63 ± 0.16	1.47 ± 0.14^a^
*T*		0.157	4.055	0.060	4.413	0.290	2.876
P		0.876	0.000	0.953	0.000	0.773	0.005

Note: Compared with pre-treatment.

^a^*p <* 0.05.

### Comparison of complication rates

The complication rate in the SG (6.98%) was lower than that in the CNG (25.58%) (*p <* 0.05; Table 4).

**Table 4:** Comparison of complication rates (cases [%]).

Group	n	Incisional infection	Trocar obstruction	Acute pancreatitis	Biliary fistula	Gastrointestinal bleeding	Total
Study group	43	1 (2.33)	1 (2.33)	0	0	1 (2.33)	3 (6.98)
Control group	43	3 (6.98)	2 (4.65)	1 (2.33)	2 (4.65)	3 (6.98)	11 (25.58)
*χ^2^*							4.132
p							0.042

## Discussion

AOSC is commonly diagnosed in elderly people aged ≥ 60 years and is caused by acute obstruction of the bile ducts, preventing bile excretion. Accumulation of bile duct materials in the common bile duct causes a dramatic increase in bile duct internal pressure and progressive necrosis due to damage to the mucosal surface cells of the bile duct, and affects the blood barrier function of the liver, resulting in hepatobiliary system injury. If left untreated, a large number of bacteria will grow and multiply on the surface of necrotic cells, causing serious bacterial infection and inducing severe inflammatory reactions. Additionally, bacteria and their endotoxins can enter the blood circulation and produce biliary endotoxemia, which induces systemic infection, sepsis, multi-organ failure, and even death in patients.^9^ Therefore, early biliary decompression, adequate bile drainage, unobstructed biliary drainage, and active anti-infection therapy are the principles of treatment for AOSC combined with septic shock.

Biliary decompression is an effective method of relieving biliary obstruction. Currently, the methods of biliary decompression include traditional choledochotomy with T-tube drainage, ERCP plus ENBD, and PTCD. Since the condition of patients is critical if radical surgery is performed at that time, it will cause a double blow to the patient, which will aggravate the trauma and disturbance of the internal environment; in serious cases, it may cause grave complications. Therefore, it is recommended to perform ERCP plus ENBD or PTCD first and then perform surgery 1–3 months after the inflammation has subsided.^10^ ERCP can lead to various serious complications, particularly acute pancreatitis, which limits its widespread use to a certain extent. The main causes of pancreatitis are the spread of infection during imaging, excessive pressure, excessive numbers of intubations, multiple injections in the pancreatic duct with accidental injury or excessive burns to the opening of the pancreatic duct and surrounding mucosa, an incision or failure to remove stones, and ampullary obstruction. Patients with underlying pancreatic disease are more likely to develop acute pancreatitis after ERCP than those without pancreatic disease. Patients with Oddi sphincter dysfunction, inflammatory strictures, and bile duct manometry are prone to postoperative pancreatitis. Endoscopic nasobiliary drainage is a minimally invasive treatment for early biliary duct decompression and drainage and is performed under the direct view of a duodenoscope. It cannot only relieve the obstruction of the bile and pancreatic ducts and achieve the effect of unobstructed drainage, but also effectively reduce the pressure on the bile and pancreatic ducts for intrahepatic and bile duct silt-like calculi and biliary pancreatitis, reduce the symptoms of poisoning, and play a therapeutic role, so that the patient's condition can be rapidly relieved, shortening the hospitalization time and reducing the treatment cost, which has obvious social benefits. Therefore, nasobiliary drainage is performed after ERCP. However, ERCP plus ENBD requires high patient tolerance, and when the lower segment is severely stenosed, placement is difficult and prone to failure. There are also risks of acute pancreatitis, retrograde infection, and perforation.^11^^,^^12^ Ultrasound-guided PTCD is a minimally invasive procedure, and simple drainage via ultrasound-guided puncture can rapidly relieve the symptoms of biliary obstruction and infection. The ultrasound-guided PTCD procedure can promote the improvement of obstructive symptoms in patients with AOSC combined with septic shock, and the symptoms of septic shock disappeared 3‒7 days after the catheter placement without serious complications.^13^ The results of this study showed that the operative time, intraoperative blood loss, duration of fever, abdominal pain, chills, and placement and drainage in the SG were shorter than those in the CNG; the overall effective rate (88.37%) was higher than that in the CNG (69.77%), and the complication rate (6.98%) was lower than that in the CNG (25.58%), which was consistent with the above study. It is suggested that the ultrasound-guided PTCD procedure for AOSC combined with septic shock is less invasive and more effective and that patients experience faster symptom relief and less pain.

Severe biliary system infections are often associated with the abnormal expression of inflammatory factors and impairment of liver function.^14^^,^^15^ In this study, both groups of patients with AOSC and septic shock had liver function impairment before surgery, which was manifested by elevated TBIL, DBIL, AST, and ALT levels. Ultrasound-guided PTCD significantly inhibited inflammatory responses and reduced serum PCT, IL-6, and CRP levels.^16^ In this study, after treatment, the levels of liver function indexes TBIL, DBIL, AST, ALT, and levels of inflammatory factors PCT, IL-6, TNF-α, and CRP were significantly reduced in both groups and were lower in the SG than in the CNG, suggesting that both procedures can promote the recovery of liver function and significantly reduce the inflammatory response, but the ultrasound-guided PTCD procedure is more effective. The mechanism of action may be that, compared with ERCP plus ENBD, ultrasound-guided PTCD can promote the drainage of stagnant bile and purulent material more effectively, thus further reducing the inflammatory response and achieving better control of infection levels. Effective drainage reduces the pressure on the bile duct, which can reduce liver damage and gradually restore liver function.

Patients with AOSC and septic shock experience abdominal pain, and surgical trauma can cause the secretion of nociceptive factors that aggravate pain sensation.^17^ NPY can affect the production of calcium ions in the postsynaptic membrane and accelerate the contraction of blood vessels, particularly small blood vessels, causing vasospasms and pain.^18^^,^^19^ PGE2 affects peripheral injury receptors and transmits pain sensation in the spinal cord, whereas PGE2 increases the pain sensitivity of the nerve root and promotes inflammatory responses that exacerbate local swelling and pain.^20^^,^^21^ 5-HT promotes the excitation of sympathetic receptors and can promote increased secretion of amines to enhance pain sensation as well as direct or indirect stimulation of injurious receptors to produce pain sensitization.^22^^,^^23^ In this study, both groups experienced varying degrees of abdominal pain before surgery, and their serum NPY, PGE2, and 5-HT levels were high. After treatment, serum NPY, PGE2, and 5-HT levels were reduced in both groups, whereas the duration of abdominal pain in the SG was shorter than that in the CNG. It has been suggested that ultrasound-guided PTCD can promote bile drainage, relieve obstruction and pain faster, and be less traumatic and painful for patients. The results of this study also showed that after surgery, there were no statistically significant differences in CD3+, CD4+, and CD4+/CD8+ levels before and after treatment in the SG, whereas the CD3+, CD4+, and CD4+/CD8+ levels in the CNG were all significantly lower than those before treatment. After surgery, the levels of CD3+, CD4+, and CD4+/CD8+ cells in SG were significantly higher than those in CNG, suggesting that ultrasound-guided PTCD has a small impact on immune function and promotes the recovery of immune function.

This study had some limitations including the small sample size and factors affecting the quality of surgery; further studies with an expanded sample size are needed to confirm our conclusions.

## Conclusion

In conclusion, ultrasound-guided PTCD for AOSC combined with septic shock can reduce surgical trauma, improve treatment efficacy, reduce stress response, facilitate recovery of liver and immune functions, and have a low complication rate, which can assist patients in passing the critical period smoothly before surgery, avoiding the risk of emergency surgery.

## Authors’ contributions

Miao Meng and Xiaobin Peng designed the experiments. Hui Feng and Shuan Tang conducted the experiments and analyzed the experimental results. Miao Meng wrote the manuscript and Xiaobin Peng revised it. All the authors approved the final manuscript.

## Funding

This work was supported by Wuxi nº 2 People's Hospital (YZGD22).

## Conflicts of interest

The authors declare no conflicts of interest.
